# Simultaneous Channel and Feature Selection of Fused EEG Features Based on Sparse Group Lasso

**DOI:** 10.1155/2015/703768

**Published:** 2015-02-24

**Authors:** Jin-Jia Wang, Fang Xue, Hui Li

**Affiliations:** College of Information Science and Engineering, Yanshan University, Qinhuangdao 066004, China

## Abstract

Feature extraction and classification of EEG signals are core parts of brain computer interfaces (BCIs). Due to the high dimension of the EEG feature vector, an effective feature selection algorithm has become an integral part of research studies. In this paper, we present a new method based on a wrapped Sparse Group Lasso for channel and feature selection of fused EEG signals. The high-dimensional fused features are firstly obtained, which include the power spectrum, time-domain statistics, AR model, and the wavelet coefficient features extracted from the preprocessed EEG signals. The wrapped channel and feature selection method is then applied, which uses the logistical regression model with Sparse Group Lasso penalized function. The model is fitted on the training data, and parameter estimation is obtained by modified blockwise coordinate descent and coordinate gradient descent method. The best parameters and feature subset are selected by using a 10-fold cross-validation. Finally, the test data is classified using the trained model. Compared with existing channel and feature selection methods, results show that the proposed method is more suitable, more stable, and faster for high-dimensional feature fusion. It can simultaneously achieve channel and feature selection with a lower error rate. The test accuracy on the data used from international BCI Competition IV reached 84.72%.

## 1. Introduction

Brain-computer interfaces (BCIs), which are communication systems designed to transmit information between the brain and computers or other electronic devices, are currently the most popular technique used in neurological rehabilitation [1]. The system does not depend on the brain's normal pathways of peripheral nerves and muscles but relies on signal acquisition technology to capture the signal generated from brain activity, which is used to control external equipment after analysis and processing. The electroencephalogram (EEG) signal is the brain signal that is obtained by noninvasive electrode acquisition. EEG signal feature extraction and classification have become a hot topic in BCI research.

The biggest problem of BCIs based on EEG signals is the high dimensions of the EEG feature space and the limited number of samples. This has prompted research into EEG channel selection and BCI feature selection. Research into feature selection and channel selection of the EEG signal can be roughly divided into two types. The first type is feature selection methods. Coelho introduced a new artificial immune network algorithm to realize automatic feature selection using the EEG signal power spectral density feature, which used an extreme learning machine as a classifier [2]. Rejer used blind source separation, a genetic algorithm and a forward feature selection method [3, 4]. Bhattacharyya proposed a differential evolution and mimetic algorithm for high-dimensional EEG signal power spectrum density feature selection [5]. Noshadi proposed an algorithm which combines Lempel–Ziv with EMD for feature extraction on the EEG signal, using* t*-test and a forward or backward feature selection method [6]. The second type is EEG signal channel selection methods. Arvaneh proposed a sparse common spatial pattern algorithm and a robust sparse common spatial pattern algorithm for channel selection. The classification results are better than the feature selection method based on Fisher criterion, mutual information, support vector machine, and common spatial pattern or a regularized common spatial pattern [7, 8]. He proposed a genetic algorithm for feature selection based on the maximized Rayleigh coefficient feature [9]. Yang proposed a method for channel selection of a specific object based on Fisher discriminant analysis scoring criteria. This method can effectively reduce the number of channels from 118 channels to no more than 11 without significantly reducing the classification accuracy by shortening the training time [10]. Gonzalez proposed a combination of Fisher discriminant analysis and a multiobjective real/binary hybrid particle swarm algorithm, which can maximize the classification accuracy and minimize the number of channels while searching for EEG channels and the classifier parameters [11]. As can be seen, most studies undertake research on EEG signals by either feature selection or channel selection unilaterally.

Lasso (least absolute shrinkage and selection operator) is a new regularization method which can be used to select high-dimensional features [12]. Group Lasso is an extended Lasso method [13], while Sparse Group Lasso is a regularization method which combines Lasso and Group Lasso [14, 15]. Germán et al. proposed a Lasso feature selection method based on minimum angle regression using fusion characteristics of the EEG, such as the power spectrum, Hjorth parameters, AR model coefficients, and wavelet transform parameters. This method used linear discriminant analysis as the classifier [16]. Experimental results show that this method is superior to traditional methods. Yeh studied the image classification problem of audio and video, using the fusion of the Mel-frequency cepstral coefficients (MFCC) feature, scale invariant feature transform (SIFT) descriptor subfeatures, histogram of oriented gradients (HOG) descriptor subfeatures, Gabor texture features, and edge direction histogram (EDH) described characteristics, and then proposed a multicore learning framework which is based on Group Lasso for feature selection [17]. Xie studied the problem with selection of uncertainty characteristics based on Sparse Group Lasso for data mining and has done experiments on nine types of UCI machine learning datasets [18].

**Figure 1:**
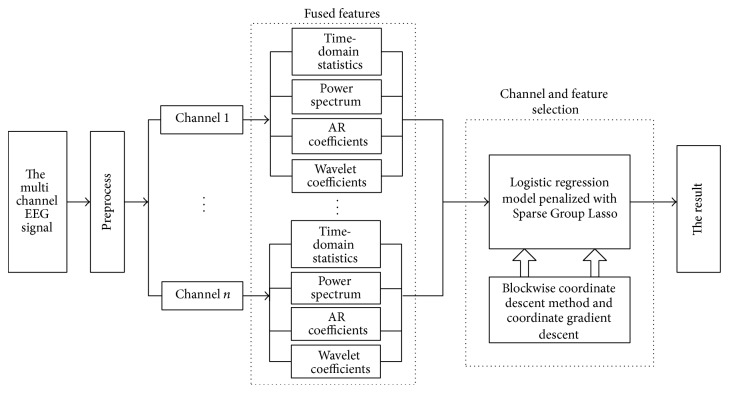
System diagram of the proposed method.

Based on the literature [16], this paper proposes the Sparse Group Lasso method for channel selection and feature selection of the EEG fused feature and estimates model parameters using a combination of the blockwise coordinate descent method and the coordinate gradient descent method. This has the ability to not only select features between channels but also select features within the channel and achieves high-dimensional EEG signal channel selection and feature selection simultaneously, while obtaining better sparse performance and classification accuracy. We conduct experimental verification on dataset 1 of the international BCI Competition IV. The EEG data is firstly preprocessed and then fused features are established from each channel of the multichannel signal; that is, the power spectrum, time-domain statistics, autoregression (AR) model coefficient, and wavelet features are extracted. The wrapped channel and feature selection method is then used. The logistic regression model is penalized with the Sparse Group Lasso to fit the training data, and parameter estimation is obtained using the blockwise coordinate descent method and coordinate gradient descent method. Finally, the test sample is classified using the trained model. The method proposed in this paper includes feature fusion, channel selection, and feature selection, as shown in Figure 1.

## 2. Feature Extraction

In study of EEG signal classification problems, an important factor in improving the recognition rate is to extract representative features to represent the EEG signal properly. In this paper, in order to extract the EEG signal features and establish high-dimensional feature fusion comprehensively, we jointly apply four types of feature extraction methods: frequency-domain analysis, time-domain analysis, analysis of time and space, and time-frequency analysis.

Power spectrum estimation can analyze the distribution and change in EEG signal rhythm [19] and capture the event-related desynchronization (ERD) and event-related synchronization (ERS) which is closely associated with movement consciousness. In our experiment, we extract the band-pass power for the time series of each channel signal as the features, for five frequency bands. The frequency bands are 2–4 Hz, 4–8 Hz, 8–12 Hz, 12–18 Hz, and 18–30 Hz, respectively.

For the time sequence of each channel of the EEG signal, we extract four commonly used statistical features: the mean value and standard deviation of the time sequence and the mean value of the first difference absolute value and the second difference absolute value of the time sequence.

The AR model is an effective tool for time sequence modeling and it has been widely used in BCI systems [20]. In our experiment, we establish a sixth order AR model for the time sequence of each channel and take the coefficient of the model as a feature of the EEG signal.

Wavelet transform is a type of variable resolution time-frequency analysis method; it has good localization in the time-domain and frequency-domain and is used for EEG signal feature extraction frequently. We use the Db4 wavelet as the mother wavelet in the experiment, make six decompositions of the time sequence of each channel, take the energy of the approximate coefficients and detail coefficients (seven-dimensional) as features, and extract four features for each of them: the Shannon entropy, logarithmic energy entropy, and the mean value and variance of the Teager-Kaiser energy operator. This constitutes 55-dimensional wavelet features overall.

## 3. Channel Selection and Feature Selection

The feature extraction process described above is carried out for each channel in the time series. While tasks to imagine different movements activate different brain areas, not all regions of the brain's electrical activity are associated with each task, so the fused features established using every channel of the EEG signal have some redundancy. Hence, we need to complete channel selection and feature selection. Channel selection removes channels which are not related to the category of imagined movement. In addition, some of the features have nothing to do with classes other than the category of imagined movement, so feature selection is required. Feature selection considers whether each dimension's characteristic is associated with each category of imagined movement, and selections are made based on the features rather than the channel.

**Figure 2:**
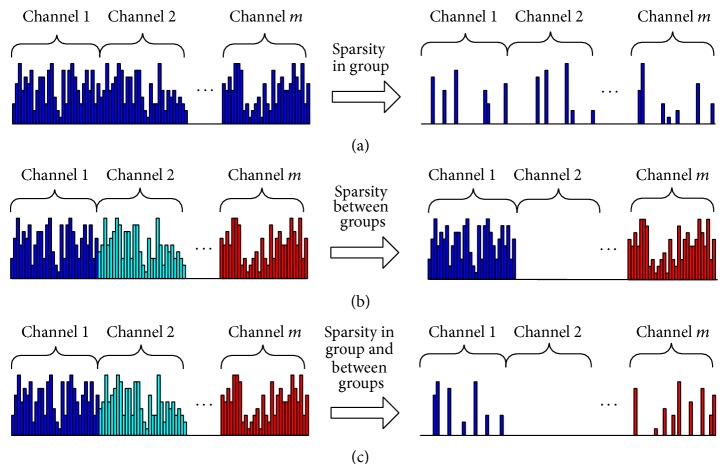
Diagram of channel selection and feature selection.

It is well known that the Lasso method can obtain a sparse solution from high-dimensional data. For feature fusion, the method extracts characteristics from each different channel without distinction, adopting the same selection standards, and can realize the process of feature selection, as shown in Figure 2(a). However, the method does not significantly reduce the number of channels. The Group Lasso method regards the fused features extracted from each individual channel as a feature set, and selection is made on a channel basis; that is, all characteristics of the channel are retained or discarded, as shown in Figure 2(b). However, with feature fusion, not all features extracted from a channel are necessarily associated with imagined movement categories, and therefore feature selection within the channel is needed. Therefore, a method is required to increase the sparsity of the feature set among channels and within each channel. The Sparse Group Lasso method is a combination of Lasso and Group Lasso, which can achieve sparsity between groups and within the group. Therefore, in this paper we propose the Sparse Group Lasso method to solve the problem of channel selection and feature selection for EEG signal feature fusion, as shown in Figure 2(c). Additionally, we propose a method that combines the blockwise coordinate descent and coordinate gradient descent to estimate the parameters of the Sparse Group Lasso model, where nonzero model parameters signify that the corresponding feature or feature group is selected and vice versa.

First, we provide the proposed logistic regression multiclassification model penalized with the Sparse Group Lasso of the EEG signal. The method can be described as follows: we assume that the training sample set is (*x*_*i*_, *g*_*i*_),  *i* = 1,…, *N*, *x*_*i*_ ∈ *R* ^*p*×*d*^ is the observation vector, *p* is the number of channels, and *d* is the dimension of each channel. We let *g*_*i*_ denote the multiclass response, *g*_*i*_ ∈ {1, 2,…, *M*}. The EEG data used in this paper is two-class data, but in order to make the algorithm more general in our description here, we give an example using a multiclassification model. The logistic regression model is used to represent the conditional probability; then the probability of sample *i* belonging to class *m* is described as

(1) $$\begin{array}{c} p_{i,m}≜P\left(g_{i}=m\;|\;x_{i}\right)=\;\frac{\exp\left(x_{i}\beta_{\cdot m}\right)}{\sum_{l=1}^{M}\exp\left(x_{i}\beta_{\cdot l}\right)}\;m=1,\ldots ,M. \end{array}$$

Here, *β* is the coefficient matrix, which represents the model parameters which need to be solved, and *β*_·*m*_ is the *m*th column of *β*. We let *g* = *M* as a reference, and then we can obtain *M* − 1 logistic models:

(2) $$\begin{array}{c} \ln\frac{P\left(g_{i}=m\;|\;x_{i}\right)}{P\left(g_{i}=M\;|\;x_{i}\right)}=x_{i}\beta_{\cdot m}\;m=1,\ldots ,M-1. \end{array}$$

Here, *x*_*i*_ *β*_·*M*_ = 0.

Using maximum likelihood estimation to fit the model, we define matrix *Y* with elements as follows:

(3) $$\begin{array}{c} y_{i,m}=\left\{\begin{array}{cc} 1 & g_{i}=m\\ 0 & g_{i}\neq m. \end{array}\right. \end{array}$$

The training dataset can be considered as *N* independent observations to simplify calculations. We take the logarithm likelihood function as follows:

(4) lβ=∑i=1  N∑m=1Myi,mln⁡pi,m=∑i=1N∑m=1Myi,mxiβ·m−ln⁡∑l=1Mexp⁡xiβ·l.

After adding a Sparse Group Lasso penalty function to (4), the objective function becomes

(5) min⁡β−lβ+λΦ(β)β =min⁡−lβ+λ1−α∑J=1pβJ2+λα∑j=1p×d×Mβj.

Here, *λ* > 0 and when *λ* is sufficiently large, **β** is zero, *α* ∈ [0,1].

 
**β**^(*J*)^ is the *J*th group of **β**, which represents the coefficient vector of the *J*th channel fused feature of each class, with dimension *d*, *J* = 1,…, *p*.
 
*β*_*j*_^(*J*)^ is the*j*th feature coefficient of the *J*th group of **β**, *j* = 1,…, *d* × *M*.
 
*β*_*j*_ is the *j*th feature coefficient of **β**, *j* = 1,…, *p* × *d* × *M*.

We can see that the Sparse Lasso penalty is a combination of Group Lasso penalty and Lasso penalty, and when *α* = 0 or *α* = 1, it converts to Group Lasso or Lasso estimation, respectively.

As described in a previous study [21], the model parameter estimation algorithm proposed in this paper is composed of three main loops: an outer coordinate gradient descent loop (Algorithm 1), a middle blockwise coordinate descent loop (Algorithm 2), and an inner modified coordinate descent loop (Algorithm 3).

For Algorithm 1, we let *f*(**β**) = −ℓ(**β**), *q* = ∇*f*(**β**), and **H** = ∇^2^ *f*(**β**), where **H** is the Hessian matrix of *f*(**β**). Then the quadratic approximation of *f*(**β**) at point $\tilde{\beta }$ is

(6) fβ=qTβ−β~+12β−β~THβ−β~+fβ~+Oβ=Qβ−qTβ~+12β~THβ~+fβ~+Oβ.

Here, $Q\left(\beta \right)={\left(q-H\tilde{\beta }\right)}^{T}\beta +(1/2)\beta^{T}H\beta$. Ignoring the irrelevant items in (6), (5) can be simplified as

(7) $$\begin{array}{c} \underset{\beta \in R^{p}}{\min}Q\left(\beta \right)+\lambda \Phi \left(\beta \right). \end{array}$$

**Algorithm 1 (outer loop used in the model parameter estimation algorithm).**

 Coordinate gradient descent
   Input: $\tilde{\beta }=\beta_{0}$
   Iterate until convergence occurs
   Let $q=\nabla f\left(\tilde{\beta }\right)$, $H=\nabla^{2}f\left(\tilde{\beta }\right)$, $Q\left(\beta \right)={\left(q-H\tilde{\beta }\right)}^{T}\beta +\left(1/2\right)\beta^{T}H\beta$
   Compute $\hat{\beta }=\min_{\beta \in R^{p\times d\times M}}Q\left(\beta \right)+\lambda \Phi \left(\beta \right)$
   Compute the step size *t* and set $\tilde{\beta }=\tilde{\beta }+t\Delta$, for $\Delta =\tilde{\beta }-\hat{\beta }$.

The purpose of Algorithm 2 is to solve the quadratic optimization problem in (7). Since the penalty Φ is separable, (7) can be written as

(8) $$\begin{array}{c} Q\left(\beta \right)+\lambda \overset{p}{\underset{J=1}{\sum }}\Phi^{\left(J\right)}\left(\beta^{\left(J\right)}\right). \end{array}$$

Here, we can use the blockwise coordination descent algorithm because Φ^(*J*)^ is convex. Taking the *J*th group (the fused feature coefficients of the *J*th channel), the problem can be simplified to

(9) $$\begin{array}{c} \underset{{\hat{\beta }}^{\left(J\right)}\in R^{p_{J}}}{\min}Q^{\left(J\right)}\left({\hat{\beta }}^{\left(J\right)}\right)+\lambda \Phi^{\left(J\right)}\left({\hat{\beta }}^{\left(J\right)}\right). \end{array}$$

Here, ${\hat{\beta }}^{\left(J\right)}$ represents the estimation of the *J*th group.

Since **H** is a diagonal matrix, it can be broken down into block matrices of *m* × *m* size. By symmetry of **H**, we obtain

(10) QJβ^J =β^JTq−Hβ~J+122∑Iβ^JTHIJβI−β^JTHJJβJ =β^JTqJ+Hβ−β~J−HJJβJ+12β^JTHJJβ^J.

Equation (10) can be rewritten as

(11) $$\begin{array}{c} Q^{\left(J\right)}\left({\hat{\beta }}^{\left(J\right)}\right)={\hat{\beta }}^{\left(J\right)T}g^{\left(J\right)}+\frac{1}{2}{\hat{\beta }}^{\left(J\right)T}H_{JJ}{\hat{\beta }}^{\left(J\right)}. \end{array}$$

Here, **g** ^(*J*)^ is the group gradient and $g^{\left(J\right)}=q^{\left(J\right)}+{\left[H\left(\beta -\tilde{\beta }\right)\right]}^{\left(J\right)}-H_{JJ}\beta^{\left(J\right)}$. ${\hat{\beta }}^{\left(J\right)}=0$ when $\sqrt{d\times M\times \left(\lambda \alpha ,g^{\left(J\right)}\right)}\leq \lambda \left(1-\alpha \right)$.

**Algorithm 2 (middle loop used in the model parameter estimation algorithm).**

 Blockwise coordinate descent
   Iterate until convergence occurs
   Choose the next block index *J* according to the cyclic rule
   Compute the block gradient **g** ^(*J*)^
   if $\sqrt{d\times M\times \left(\lambda \alpha ,g^{\left(J\right)}\right)}\leq \lambda \left(1-\alpha \right)$, then let **β** ^(*J*)^ = 0
   else
   
$\beta^{\left(J\right)}=\min_{{\hat{\beta }}^{\left(J\right)}\in R^{p_{J}}}Q^{\left(J\right)}\left({\hat{\beta }}^{\left(J\right)}\right)+\lambda \Phi^{\left(J\right)}\left({\hat{\beta }}^{\left(J\right)}\right)$.

For Algorithm 3, we rewrite (9) as

(12) $$\begin{array}{c} \underset{\text{loss}}{\underset{︸}{Q^{\left(J\right)}\left({\hat{\beta }}^{\left(J\right)}\right)+\lambda \left(1-\alpha \right){\left\|{\hat{\beta }}^{\left(J\right)}\right\|}_{2}}}+\underset{\text{penalty}}{\underset{︸}{\lambda \alpha \overset{p_{J}}{\underset{j=1}{\sum }}\left|{\hat{\beta }}_{j}^{\left(J\right)}\right|}}. \end{array}$$

The two first terms of (12) are considered to be the loss function and the last term is the penalty. The loss is not differentiable at zero due to the L2-norm, and thus we cannot completely separate out the nondifferentiable parts, so the coordinate descent method has been modified for this case. For the *j*th iteration of the *J*th group, we need to find the minimum of the function $\omega \left({\hat{\beta }}_{j}^{\left(J\right)}\right)$:

(13) $$\begin{array}{c} \omega \left({\hat{\beta }}_{j}^{\left(J\right)}\right)=c{\hat{\beta }}_{j}^{\left(J\right)}+\frac{1}{2}h{\hat{\beta }}_{j}^{\left(J\right)2}+\gamma \sqrt{{\hat{\beta }}_{j}^{\left(J\right)2}+r}+\xi \left|{\hat{\beta }}_{j}^{\left(J\right)}\right|, \end{array}$$

where *c* = *g*_*j*_^(*J*)^ + ∑_*k*≠*j*_(**H**_*JJ*_)_*jk*_ *β*_*k*_^(*J*)^, *γ* = *λ*(1 − *α*), *r* = ∑_*k*≠*j*_ *β*_*k*_^(*J*)2^, *ξ* = *λα*, and *h* is the *j*th diagonal of the Hessian block **H**_*JJ*_.

Due to the convexity of *f*(**β**), we conclude that *h* ≥ 0. Since the quadratic approximation *Q*(**β**) is bounded by the constraints below, we obtain ${\hat{\beta }}_{j}^{\left(J\right)}=0$ when *h* = 0. When *h* > 0, ${\hat{\beta }}_{j}^{\left(J\right)}$ can be obtained as follows.

If *r* = 0 or *γ* = 0, then

(14) $$\begin{array}{c} {\hat{\beta }}_{j}^{\left(J\right)}=\left\{\begin{array}{cc} \frac{\xi +\gamma -c}{h} & \text{if}\;\;c>\xi +\gamma \\ 0 & \text{if}\;\;\left|c\right|\leq \xi +\gamma \\ \frac{-\xi -\gamma -c}{h} & \text{if}\;\;c<-\xi -\gamma . \end{array}\right. \end{array}$$

If *r* > 0, *γ* > 0, and |*c*| ≤ *ξ*, then ${\hat{\beta }}_{j}^{\left(J\right)}=0$ and therefore

(15) $$\begin{array}{c} c+\mathrm{sgn}\left(\xi -c\right)\xi +h{\hat{\beta }}_{j}^{\left(J\right)}+\gamma \frac{{\hat{\beta }}_{j}^{\left(J\right)}}{\sqrt{{\hat{\beta }}_{j}^{\left(J\right)2}+r}}=0. \end{array}$$

We solve (15) by applying a standard root finding method. We define Δ ∈ *R* ^*p*×*d*×*M*^ and can then rewrite the descent direction at zero for function (12):

(16) $$\begin{array}{c} \Delta_{i}^{\left(J\right)}=\left\{\begin{array}{cc} 0 & \left|g_{i}^{\left(J\right)}\right|\leq \lambda \alpha \\ g_{i}^{\left(J\right)}-\lambda \alpha \mathrm{sgn}\left(g_{i}^{\left(J\right)}\right) & \text{else}. \end{array}\right. \end{array}$$

**Algorithm 3 (inner loop used in the model parameter estimation algorithm).**

 Modified coordinate descent scheme
   Iterate until convergence occurs
   Choose the next parameter index *j* according to the cyclic rule
   Compute *β*_*j*_^(*J*)^ using (14) and  (15)
   If ‖**β** ^(*J*)^‖_2_ < *ε* and *Q* ^(*J*)^(**β** ^(*J*)^ + *λ*Φ^(*J*)^(**β** ^(*J*)^)) ≥ 0 then
   Compute the descent direction Δ^(*J*)^ of (12) at zero by  (16)
   Use line search to find *t*, such that *Q* ^(*J*)^(*t*Δ^(*J*)^) + *λ*Φ^(*J*)^(*t*Δ^(*J*)^) < 0
   Let **β** ^(*J*)^ = *t*Δ^(*J*)^.

## 4. Experimental Process and Results Analysis

### 4.1. Description of Data

**Figure 3:**
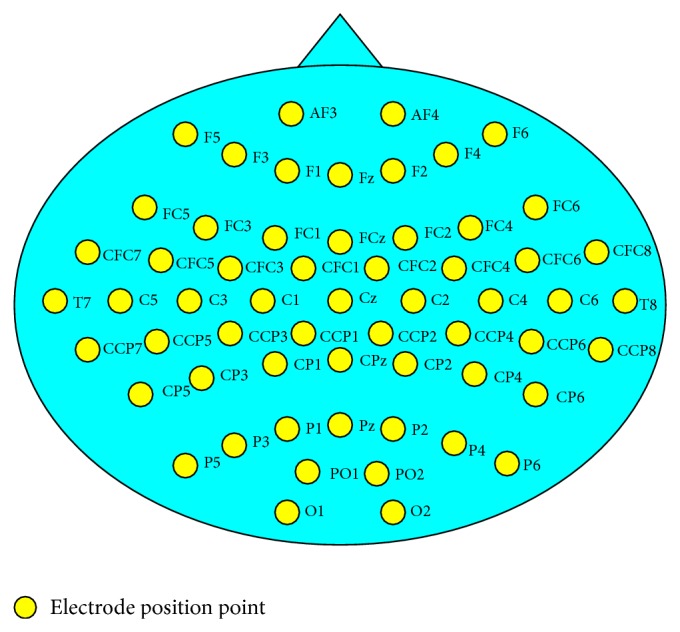
Schematic diagram of 59 channels of the BCI.

The EEG data from international BCI Competition IV dataset 1 was used in experiments, as shown in Table 1. The four datasets were recorded from 4 healthy subjects and sampled from 59 electrodes which were distributed over the head, with each dataset containing 400 sampling points. Within the four groups of data, datasets A and F are imagined movement data for the left hand and foot, and datasets E and G are imagined movement data for the left and right hand. The original data used 59 electrodes, with a sampling frequency of 1000 Hz, and the duration of each experiment was 4 s. We resampled the signal with a sampling frequency of 100 Hz.  Figure 3 shows the schematic diagram of the 59 channels in the brain-machine interface. The first channels 1–10 in the EEG signal acquisition channels are, respectively, “AF3,” “AF4,” “F5,” “F3,” “F1,” “Fz,” “F2,” “F4,” “F6,” and “FC5,” channels 11–20 are, respectively, “FC3,” “FC1,” “FCz,” “FC2,” “FC4,” “FC6,” “CFC7,” “CFC5,” “CFC3,” and “CFC1,” channels 21–30 are, respectively, “CFC2,” “CFC4,” “CFC6,” “CFC8,” “T7,” “C5,” “C3,” “C1,” “Cz,” and “C2,” channels 31–40 are, respectively, “C4,” “C6,” “T8,” “CCP7,” “CCP5,” “CCP3,” “CCP1,” “CCP2,” “CCP4,” and “CCP6,” channels 41–50 are, respectively, “CCP8,” “CP5,” “CP3,” “CP1,” “CPz,” “CP2,” “CP4,” “CP6,” “P5,” and “P3,” and channels 51–59 are, respectively, “P1,” “Pz,” “P2,” “P4,” “P6,” “PO1,” “PO2,” “O1,” and “O2.”

### 4.2. Experimental Process and Results Analysis

The first step was to extract the features from the *p* = 59 channels of the EEG signal. For each channel signal, a 5-dimensional power spectral feature, 4-dimensional time-domain statistical feature, 6-dimensional AR model coefficient feature, and 55-dimensional wavelet decomposition coefficient feature were extracted. Thus, the fused features of each channel were 70-dimensional.

In this paper, the Sparse Group Lasso method was firstly used for EEG signal processing. The features of each channel were extracted as a group, that is, *β* ^(*J*)^, where *J* = 1,…, *p*, with *p* = 59 groups in total. Here, *β* = (*β* ^(1)^,…, *β* ^(*J*)^,…, *β* ^(*p*)^), where *β* ^(*J*)^ ∈ *R* ^*d*×*M*^. In experiments, we used the wrapped Sparse Group Lasso method for channel and feature selection. At first, the feature set consisted of features extracted from each channel of the EEG signal. The combined coordinate gradient descent method and blockwise coordinate method were then used to solve the objective function with a corresponding penalty term to get the parameter estimation results, based on the training data logistic regression model. A 10-fold cross-validation method was applied to select the parameter estimation with the highest training accuracy rate as a result of channel and feature selection. Finally, the test data corresponding to the selected channel and feature subset for the trained model under test was used to calculate the test error rate.

The first experiments use datasets A and E as follows. For dataset A, the method proposed in this paper can be compared to a type of feature extraction method and feature fusion method, respectively, with results shown in Table 2.

**Figure 4:**
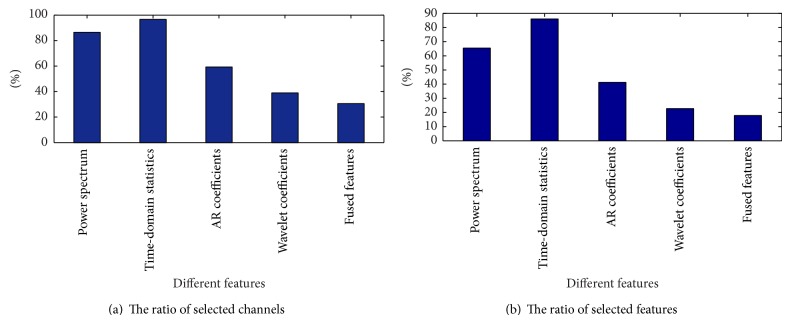
Comparison of single features on the number of selected channels and features of dataset A.

**Figure 5:**
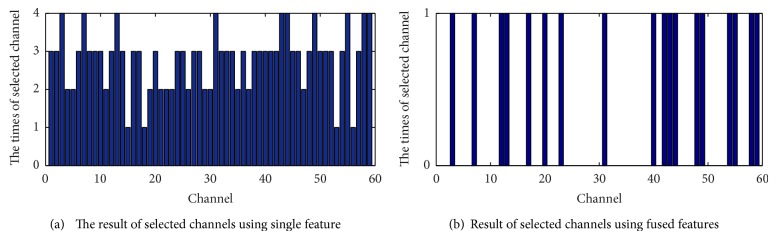
The comparison on selected channels of dataset A.

**Figure 6:**
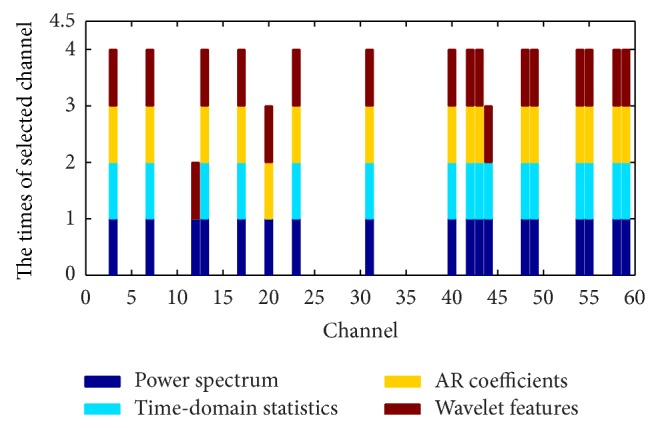
The selected channels using fused features of dataset A.

From Table 2 it can be observed that, compared with the AR coefficient and wavelet coefficient features, the feature fusion obtains a lower error rate for simultaneous channel and feature selection. For the power spectrum characteristic and the time-domain statistics characteristic, although the feature fusion error rate is slightly higher, the feature fusion method has obvious advantages for channel selection. Therefore, it can be concluded that when considering comprehensive performance of the test error rate and channel selection number, a fused feature extraction method is better than a single feature extraction method. Figures 4 and 5 compare single feature extraction and feature fusion of the channel/feature selection for dataset A, respectively, and Figure 6 shows the fused feature channel selection result analysis.

Figure 4 shows that the ratio of the number of channels and features selected is lowest when using feature fusion and it better reduces the redundancy of channel and feature.

Figure 5 shows that, out of the 18 channels selected by the feature fusion method, 15 channels are included in the selection results from three or more extraction methods, a percentage of 83.33%. In addition, there are 10 channels, F2, F5, FCz, C4, CP1, CP3, P5, P6, O1, and O2, respectively, selected by all four feature extraction methods, and these channels are important for classification of dataset A. Finally, the proposed method includes all of these channels, which indicates that the feature fusion is superior at removing redundant channels and choosing the most relevant channels for signal classification.

As an example, we observe that the 12th (FC1) channel in Figure 6 only contains the power spectrum feature and the wavelet feature; that is, only these features contribute to the classification problem from the four types of heterogeneous feature of this channel. From analysis of all 18 channels, it can be observed that selection frequency of the power spectrum feature and the wavelet feature is 100%, while the time-domain statistic and AR coefficient feature have a selection frequency of 88.89%. Therefore, compared with the time-domain statistic and AR coefficient feature, the power spectrum feature and the wavelet feature are more important in the classification of dataset A.

**Figure 7:**
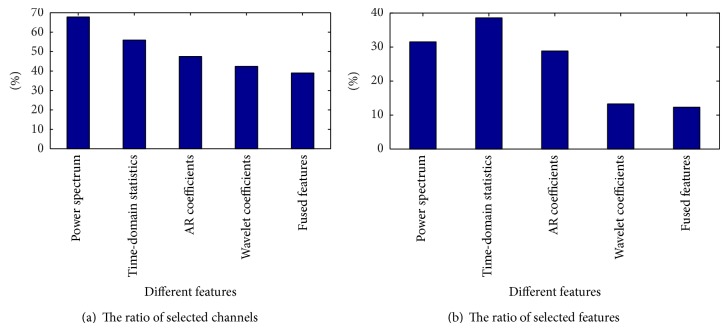
Comparison of the number of channels and features selected from dataset E.

**Figure 8:**
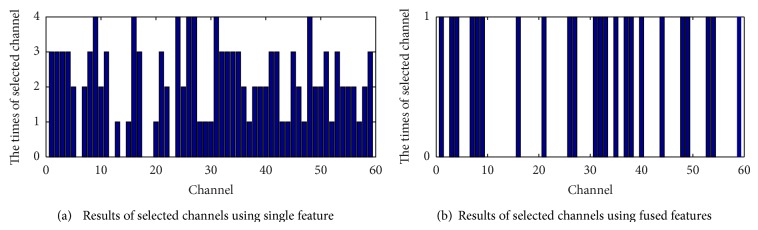
Comparison of selected channels of dataset E.

**Figure 9:**
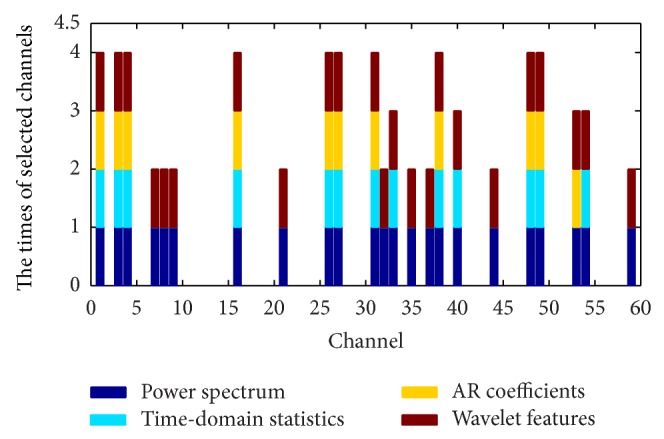
The selected channels using fused features of dataset E.

The second experiment followed the same experimental procedure and analysis for dataset E. The results are shown in Table 3 and Figures 7–9.

We can draw similar conclusions from analysis of Table 3; that is, when there is a lower or equivalent test error rate, the feature fusion method can achieve better channel and feature selection. Figures 7 and 8 compare single feature extraction and feature fusion, respectively, for the channel and feature selection of dataset E.  Figure 9 is the fused feature channel selection result analysis.

From Figure 7, we can directly observe that the fused feature extraction method achieves better dimensional reduction on the selected number of channels and features. In Figure 8, 23 channels are selected by feature fusion, with 16 channels contained in the selection results from three or more extraction methods, a percentage of 69.6%. Seven of the channels (F6, FC6, CFC8, C5, C3, C4, and CP6) are selected by all four feature extraction methods, and of these six channels (all except CFC8) are selected by the feature fusion method, a percentage of 85.7%. This shows that the feature fusion method can more accurately choose channels which are relevant to the classification.

Figure 9 shows that 23 channels (which all include the power spectrum characteristic and wavelet feature) are selected by feature fusion, 13 channels include the time-domain statistic, and 11 channels contain the AR coefficient features. From this, we conclude that the power spectrum characteristics and wavelet feature play a more important role for classification.

The above experiments have shown that the feature fusion extraction method can provide alternative features for Sparse Group Lasso. It is suitable for handling data with high dimensions and can select the most effective features from the data.

The third experiment is as follows. In the following experiments, the feature fusion extraction method is adopted. The comparative results of Lasso feature selection, Group Lasso channel selection, and Sparse Group Lasso channel and feature selection for dataset A are shown in Table 4.

**Figure 10:**
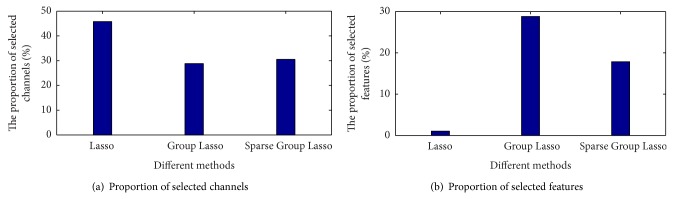
Results of three-feature channel or feature selection methods.

From Table 4 we can see that, compared with the Lasso and Group Lasso, Sparse Group Lasso can guarantee a lower error rate. Sparse Group Lasso selects more characteristics than the Lasso method but chooses a lower number of channels. Since the four datasets were collected from 59 electrodes, and each electrode corresponds to an individual channel for experiments, the channel selection represents the selection of an electrode. As each channel contains 70 characteristics, removing channel redundancy has more significance than removing redundant features. So, Sparse Group Lasso can be used for channel selection and feature selection at the same time with lower error rates.  Figure 10 compares different channel and feature selection methods for dataset A.

**Figure 11:**
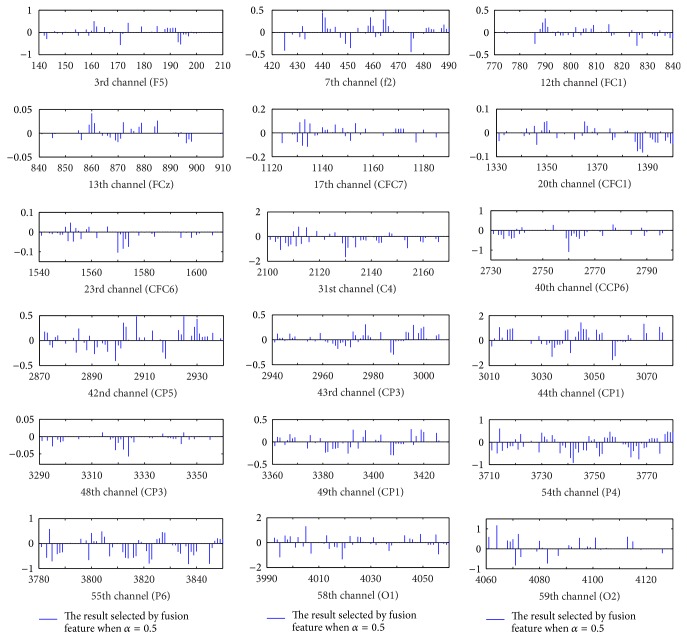
Channel and feature selection results of fused features for *α* = 0.5.

Figure 11 shows the channels and characteristics when parameter *α* = 0.5 on dataset A. Each channel is composed of 70-dimensional features: the power spectrum characteristics are 5-dimensional, the time-domain statistical features are 4-dimensional, the AR model coefficient characteristics are 6-dimensional, and the characteristics of the wavelet decomposition coefficient are 55-dimensional. As can be seen in Figure 11, 18 channels are selected from the full range of channels, and not all features are selected. For example, on the 12th channel, no features are selected between the 775th dimension and the 785th dimension. AR coefficients and time-domain statistics characteristics are stored within this interval, therefore we can determine that the 12th channel does not choose the time-domain statistics and AR coefficient characteristics (also this can be concluded from Figure 6), and similar findings can be observed through further channel analysis. Therefore, it is more intuitive to discover the sparsity between channels and within each channel and then obtain the important features. This further proves that the Sparse Group Lasso method can realize channel and feature selection at the same time.

For the fourth experiment, we compared the performance of Lasso, Elastic Net, Group Lasso, and Sparse Group Lasso for feature selection and the classification problem. The results are shown in Table 5. We present the number of selected channels and features based on Sparse Group Lasso with different values of parameter *α*  (0, 0.25, 0.5, 0.75, 1). Sparse Group Lasso is equivalent to Group Lasso when *α* equals 0 and is equivalent to Lasso when *α* equals 1. Group Lasso shares the same grouping method as Sparse Group Lasso, which takes the fused features of each channel as a group and trades off on the group level in order to make the channel selection. Lasso and the Elastic Net method treat the features extracted from all channels equally and compromise on the feature level in order to make the feature selection. We use the packaging method with fused features in the experiment on the four datasets based on Lasso feature selection, Elastic Net feature selection, Group Lasso channel selection, and Sparse Group Lasso channel and feature selection separately. As shown in Table 5, for different datasets, the larger *α* is, the lower test error rate becomes. We can conclude that the Sparse Group Lasso method can obtain the lowest error rate when making channel and feature selection when the parameter setting is close to Lasso (*α* = 1).

Table 5 shows the results of different channel/feature selection methods (Lasso, Elastic Net, Group Lasso, and Sparse Group Lasso) for different datasets. We can observe that, compared with other methods, Sparse Group Lasso obtains the lowest error rate, with the lowest number of selected channels, below 38.98% of the total number of channels for all datasets. The lowest number of selected channels is only 23.73% of the total, which reduces the redundancy of channels significantly. Since channel selection is equivalent to the choice of electrode, it has greater significance than feature selection. The number of features selected by Sparse Group Lasso is below 17.85% for all datasets, with the lowest only 7.97% of the total number of features. We conclude that the comparison shows that Sparse Group Lasso can achieve channel selection and feature selection simultaneously, while ensuring sparsity among channels and features when maintaining an error rate equal to or lower than other methods.

In comparison to other studies such as [22], we have only analyzed training sets, rather than using a testing set where the training set needs to be divided into 100 as 80% and 20% randomly. In the study in [22], 11 channels were chosen artificially: FC3, FC4, Cz, C3, C4, C5, C6, T7, T8, CCP3, and CCP4. This is different from the channels selected by our proposed method, since the previous study [22] used spatial pattern characteristics, while we use frequency-domain characteristics. The test set used, BCI Competition IV dataset 1, is continuous data, while we have piecewise processed the continuous data in order to increase the test samples. Therefore, it is not possible to compare our methods with other previous studies.

## 5. Conclusion

Classification of EEG signals is a core part of BCIs, so an effective feature extraction and selection method is the key to improving identification accuracy. For EEG signal processing, we present a new method: wrapped Sparse Group Lasso method for channel and feature selection. The joint application of a variety of feature selection methods was firstly used to establish high-dimensional feature fusion of the preprocessed EEG signals. Then, channels and features are selected in a wrapped way. The logistic regression model penalized with Sparse Group Lasso is fitted on the training data, and parameter estimation is obtained by a blockwise coordinate descent method and coordinate gradient descent method. The best feature subset is selected by using 10-fold cross-validation. Finally, the test sample is classified using the trained model, and the feature extraction method included the power spectrum, time-domain statistics, AR model, and the wavelet coefficients. Fusing multiple features to establish a collection to make a selection is a beneficial research area to explore for EEG signal classification. Experiments have shown that this method can extract the characteristics of the EEG signal more completely, so it is an effective way to improve the signal recognition accuracy. Compared with existing channel and feature selection methods, the results show that the method proposed is more suitable for selecting a subset of fused feature of the EEG signal, as well as being more stable and faster. It can also select a subset which is more relevant to the classification, and the test accuracy obtained on the data used from international BCI Competition IV reached 84.72%. This method is a good choice for future research in pattern recognition topics, such as speech recognition, face recognition, gene classification, remote sensing image recognition, and medical image recognition.

## Figures and Tables

**Table 1:** Description of data used in the experiment.

Dataset	Contest name	Task of movement imagination	Number of samples (training + testing)
A	IV-1a	Left hand and foot	200 + 227
F	IV-1f		200 + 228
E	IV-1e	Left and right hand	200 + 229
G	IV-1g		200 + 234

**Table 2:** The experimental results of different feature extraction methods for dataset A.

Feature extraction method	The experimental results		
	Number of channels	Number of features	Test error rate (%)
Power spectrum	51	193	27.76
Time-domain statistic	57	203	28.64
AR coefficient	35	146	40.97
Wavelet coefficient	23	737	30.40
Fusion feature	18	737	29.26

**Table 3:** Experimental results comparing different feature extraction methods for dataset E.

Feature extraction method	Experimental results		
	Number of channels	Number of features	Test error rate (%)
Power spectrum	40	93	14.85
Time-domain statistic	33	91	16.16
AR coefficient	28	102	17.47
Wavelet feature	25	430	19.65
Fusion feature	23	508	15.28

**Table 4:** Experimental results of three methods for dataset A.

Channel/feature selection method	Selected channel		Selected feature		Test error rates (%)
	Number	Proportion (%)	Number	Proportion (%)	
Lasso	27	45.76	43	1.04	31.72
Group Lasso	17	28.81	1190	28.81	31.72
Sparse Group Lasso	18	30.51	737	17.85	29.96

**Table 5:** Experimental results on different datasets.

Channel/feature selection method	Dataset A			Dataset E			Dataset F			Dataset G		
	Channel	Feature	Error rate (%)	Channel	Feature	Error rate (%)	Channel	Feature	Error rate (%)	Channel	Feature	Error rate (%)
Elastic Net	44	120	32.60	41	89	16.16	27	38	43.86	49	168	23.50
Lasso	27	43	31.28	38	60	15.28	27	38	43.86	22	49	23.51
Sparse Group Lasso (*α* = 0.25)	16	890	31.72	22	1193	17.03	13	742	40.96	12	868	22.22
Sparse Group Lasso (*α* = 0.5)	18	737	29.26	18	703	16.59	14	584	40.52	14	686	20.94
Sparse Group Lasso (*α* = 0.75)	17	440	32.15	23	508	15.28	14	329	40.52	14	370	20.94
Group Lasso	17	1190	31.28	26	1820	17.47	16	1120	40.35	10	700	22.22
